# Respiratory Tract Infections in Children

**DOI:** 10.3390/pathogens10121596

**Published:** 2021-12-09

**Authors:** Tobias Tenenbaum

**Affiliations:** Sana Klinikum Lichtenberg, Academic Teaching Hospital, Charité-Universitätsmedizin Berlin, 10365 Berlin, Germany; Tobias.Tenenbaum@sana-kl.de

Respiratory tract infections are a major threat, causing morbidity and mortality, especially among children. In the era of the COVID-19 pandemic, they have come into major focus in the scientific world and the public. In this Special Issue on “Respiratory Tract Infections in Children”, we present recent clinical data demonstrating the role of pathogens and pathogen interactions in the respiratory tract. This Special Issue also aims to shed light on the cellular and molecular interactions between pathogens and the host.

In the context of the COVID-19 pandemic, Rybak et al. [1] demonstrated a dramatic decrease in pediatric emergency department visits due to community-acquired pneumonia during the first lockdown period in France. Data from a French prospective surveillance system were used for the analysis. The overall reduction rate of visits was –79.7%, and that of CAP admissions was –71.3%. Overall, the data indicated a significant negative impact on the circulation of respiratory tract pathogens in the community during the lockdown phase. These data were further confirmed in international studies over the course of the pandemic.

Méndez-Echevarría et al. present prospective seroprevalence data on hospitalized and non-hospitalized children who had confirmed COVID-19 infection, and compared these with the cohabitants of infected healthcare workers [2]. Interestingly, children infected with SARS-CoV-2, which caused lower respiratory tract infection or multisystemic inflammatory syndrome, maintained a particularly positive serological response six months after the infection, whereas a significant decline in the IgG titer of asymptomatic or mildly symptomatic children was observed. Disease severity, therefore, may contribute to the persistence of the host immune response.

Rudolph et al., in their prospective clinical study, looked at the clinical and laboratory characteristics of pediatric patients with febrile seizures (FS), and correlated these findings with the identified virus type and their nasopharyngeal aspirate quantity [3]. Besides the well-known pathogens that cause febrile seizures, such as human herpes virus 6 and influenza, adenovirus and rhinovirus were dominant pathogens. They further identified a high rate of viral co-infections (32%). Family history, age, infection parameters, and virus type, as well as a high viral load, were shown to be potential contributors to the presentation of FS in children’s respiratory tract infections.

Linsen et al. aimed to elucidate whether weather conditions have a strong influence on RSV activity [4]. Climate change has been proposed as a potential determinant of future RSV-related health care utilization. This is a timely study, since we are currently observing strong RSV activity outside of the traditional winter season. The authors demonstrated that the maximum temperature and global radiation best predicted PICU admissions. Despite the long observational period of 13 years, and the large patient cohort of 2161 cases, they could not determine whether this was a potential contributing factor to the increased RSV-related PICU burden in the Netherlands. This fits well with the above-described extraordinary RSV activity observed during the current pandemic, and raised the question of which other factors may be important. Future studies will address this.

The review by Slimmen et al. focuses on the role of antigen-presenting cells (APCs) in respiratory tract infections [5]. Whether a pathogen in the nasopharynx colonizes and which factors may contribute to infection could potentially depend on APS. The authors, therefore, propose a standardized nomenclature of the different stages of carriage and infection, based on the pathogen’s position with regard to the epithelium and the amount of inflammation that is present.

Betti et al. describe the role of atypical bacterial pathogens, such as *Mycoplasma pneumoniae*, in the development of leukocytoclastic small-vessel vasculitis of the skin [6]. The authors highlight the predominant role of *Mycoplasma pneumoniae* in vasculitis patients, whereas a link to other pathogens, such as *Chlamydophila pneumoniae, Chlamydophila psittaci*, *Coxiella burnetiid*, *Francisella tularensis*, or *Legionella pneumophila*, could not be demonstrated. Most patients recovered within 3 months, without sequelae.

Perez Ortiz et al. present the fatal case of a neonate with legionellosis and further present a review of the literature [7]. The neonatal patient presented with cutaneous manifestation, sepsis, respiratory distress syndrome, and, in this context, required extracorporeal membrane oxygenation. Risk factors such as water birth or the use of air humidifiers are critically discussed as potential causes of severe disease and fatal outcome.

The appearance of macrolide-resistant M. pneumoniae (MRMP) in the community, as presented in an outbreak report at a primary school in Germany, is analyzed by Hubert et al. [8]. The authors describe a cluster of hospitalized patients with MRMP that emerged during the outbreak. The macrolide resistance gene A2058G was identified. However, whether macrolide resistance bears a potential risk for disease severity could not be answered in this study.

Lastly, Kaiser et al. present the extraordinary case of an adolescent with severe pneumonia and sepsis due to a *Dialister pneumosintes* infection [9]. This pathogen is associated with dental, periodontal or sinus infections, but systemic infections are rare. The authors illustrate the case with several radiological images, and performed a literature review of invasive and severe cases of *Dialister pneumosintes* infections. The report will help to sensitize health care specialists for this rare infection.

In summary, this Special Issue collates research on frequently and rarely detected respiratory pathogens and their role in respiratory tract infections in children.
